# Mastl/PP2A regulate Cdk1 in ooycte maturation

**DOI:** 10.18632/oncotarget.4916

**Published:** 2015-07-18

**Authors:** Deepak Adhikari, Kui Liu, Philipp Kaldis

**Affiliations:** Institute of Molecular and Cell Biology (IMCB), A*STAR (Agency for Science, Technology and Research), Singapore, Republic of Singapore; and National University of Singapore (NUS), Department of Biochemistry, Singapore, Republic of Singapore

**Keywords:** Greatwall kinase, MASTL, Cdk1, meiosis, mouse oocyte

Resumption of oocyte meiotic maturation is driven by activation of cyclin-dependent kinase 1 (Cdk1), which phosphorylates target proteins that are important for nuclear envelope breakdown, chromosome condensation, and remodelling of the actin and microtubule cytoskeleton. As a consequence, a bipolar spindle is formed and condensed chromosomes become aligned at the metaphase plate. The spindle assembly checkpoint (SAC) prevents the activation of the anaphase-promoting complex/ cyclosome (APC/C^Cdc20^) during meiosis I progression until all kinetochores come under tension [for review see: 1]. Upon the satisfaction of SAC, APC/C becomes active, and degrades cyclin B and securin. Degradation of cyclin B lowers Cdk1 activity and securin degradation activates separase, which cleaves cohesion holding sister chromatids together [2]. After cytokinesis, one set of chromosomes is retained in the oocyte and the other is segregated into the first polar body (PB).

Although Cdk1/cyclin B complexes have long been considered sufficient for driving mitosis and meiosis, recent studies in various systems have shown that the simultaneous suppression of the antagonizing protein phosphatase 2A (PP2A), which is mediated by the Greatwall (Gwl) kinase (called Mastl [microtubule-associated serine/threonine kinase-like] in mammals), is also required for mitotic entry or progression [3-5]. Although protein phosphatases have previously been implicated in the regulation of oocyte maturation, the mechanisms regulating their functions have not been studied. To uncover the role of Mastl in the meiotic cell division of mouse oocytes, we generated a novel Mastl conditional knockout mouse line and studied Mastl-null oocytes [6]. We found that meiotic resumption and progression to metaphase I in Mastl-null oocytes was indistinguishable from the control oocytes; however, extrusion of the first PBs was delayed in the mutant oocytes. Securin degradation in Mastl-null oocytes was also delayed, suggesting that Mastl is required for the timely activation of APC/C that is needed for the completion of meiosis I. However, the delay in anaphase I onset and the first PB extrusion was not caused due to an unsatisfied SAC, which was indicated by a complete dissociation of Mad2 (an essential spindle checkpoint protein) from kinetochores of Mastl-null oocytes at metaphase I [6].

Meiosis in oocytes represents a specialized cell division whereby a sharp increase in Cdk1 activity after completion of meiosis I prevents them from entering S phase and oocytes transit directly to meiosis II. Moreover, the paired sister chromatids remain condensed during the meiosis I−meiosis II transition and form typical bipolar metaphase spindles and they remain arrested at metaphase II until fertilization [7]. We found that although most of the Mastl-null oocytes completed meiosis I and extruded morphologically normal PBs with some delay, these oocytes contained distinct nuclei with decondensed chromatin and did not re-form the metaphase II spindle. Notably, most of the Mastl-null oocytes maintained central spindle microtubules between the decondensed chromatin in the PB and the oocyte nucleus [6]. In Mastl-null oocytes, we found that Cdk1 activity failed to increase after meiosis I but the PP2A activity was significantly higher than in control oocytes. Higher PP2A activity prevented the activation of Cdk1 required for MII entry because the pharmacological inhibition of PP2A activity caused the elevation of Cdk1 activity and entry into MII of Mastl-null oocytes. Surprisingly, lack of Mastl did not significantly affect the PP2A and Cdk1 activities during meiosis resumption or progression to metaphase I [6]. This result is remarkable because it was expected that the mechanism for Cdk1 activation would be conserved in meiosis I, meiosis II, and mitosis. However, our results indicate that the extent of dependency of PP2A activity on Mastl underlies the differential kinetics of Cdk1 activation during meiosis I and meiosis II.

Meiosis I in mammalian oocytes is a lengthy process, and the prometaphase of meiosis I (the stage from nuclear envelope breakdown to chromosome alignment) takes about 8 hours, which is much longer than the approximately 30 minutes of prometaphase in most mitotic cells. We found that wild type mouse oocytes progressed through prometaphase I without suppressing PP2A thus the lack of Mastl did not cause a further increase in PP2A activity during this process. At the same time, the kinetics of Cdk1 activation, meiosis resumption and prometaphase I progression were not affected in Mastl-null oocytes. However, higher PP2A activity after meiosis I in Mastl-null oocytes prevented the activation of Cdk1 and entry into meiosis II [6]. Based on our findings, we propose that we have discovered a previously unknown mechanistic difference between meiosis I and mitosis/ meiosis II. Thus, oocytes maintain higher PP2A activity during meiosis I progression to antagonize Cdk1 and thus delay the progression of this process. Such a prolonged meiosis I can be useful for preventing stabilization of erroneous kinetochore-microtubule attachments, because it was previously shown that the slow Cdk1 activation during oocyte prometaphase I delays the timing of K-MT attachment [8]. It is possible that oocytes achieve the slow prometaphase I by not involving Mastl, thereby not inhibiting PP2A activity during meiosis I. On the other hand, Mastl is essential to inactivate PP2A after meiosis I when a rapid activation of Cdk1 is required for MII entry, which is similar to the rapid Cdk1 activation in mitosis.

Thus, our results indicate that Mastl mediated PP2A inhibition is crucial for the timing of anaphase I and for the entry into meiosis II in mouse oocytes, however, it also raises many new questions. For instance, if Mastl is dispensable for prometaphase I progression, is Mastl activity normally suppressed during this process? If so, what are the possible mechanisms that inactivate Mastl during prometaphase I? Following chromosome segregation in meiosis I in Mastl-null oocytes, we found that the oocyte chromosomes were rapidly decondensed and nuclear envelope was reassembled. Thus, Mastl appears to mediate an additional surveillance mechanism after chromosome segregation in meiosis I that prevents chromosome decondensation and nuclear envelope reassembly. Similarly, how Mastl regulates APC/C in meiosis I remains unknown and will require further studies.

## References

[1] Foley EA (2013). Nature Reviews Molecular Cell Biology.

[2] Kudo NR (2006). Cell.

[3] Mochida S (2010). Science (New York, NY).

[4] Gharbi-Ayachi A (2010). Science (New York, NY).

[5] Alvarez-Fernandez M (2013). Proc Natl Acad Sci U S A.

[6] Adhikari D (2014). The Journal of Cell Biology.

[7] Jones KT (2004). Molecular Human Reproduction.

[8] Davydenko O (2013). The Journal of Cell Biology.

